# Characterizing the Sensing Response of Carbon Nanocomposite-Based Wearable Sensors on Elbow Joint Using an End Point Robot and Virtual Reality

**DOI:** 10.3390/s24154894

**Published:** 2024-07-28

**Authors:** Amit Chaudhari, Rakshith Lokesh, Vuthea Chheang, Sagar M. Doshi, Roghayeh Leila Barmaki, Joshua G. A. Cashaback, Erik T. Thostenson

**Affiliations:** 1 Center for Composite Materials, University of Delaware, Newark, DE 19716, USA; amitc@udel.edu (A.C.); smdoshi@udel.edu (S.M.D.); 2 Department of Biomedical Engineering, University of Delaware, Newark, DE 19716, USA; lokerak@udel.edu (R.L.); joshcash@udel.edu (J.G.A.C.); 3 Department of Computer and Information Sciences, University of Delaware, Newark, DE 19716, USA; vuthea@udel.edu (V.C.); rlb@udel.edu (R.L.B.); 4 Department of Mechanical Engineering, Department of Materials Science and Engineering, and Center for Composite Materials, University of Delaware, Newark, DE 19716, USA

**Keywords:** carbon nanotubes, multifunctional composites, virtual therapy, wearable sensors, smart garments

## Abstract

Physical therapy is often essential for complete recovery after injury. However, a significant population of patients fail to adhere to prescribed exercise regimens. Lack of motivation and inconsistent in-person visits to physical therapy are major contributing factors to suboptimal exercise adherence, slowing the recovery process. With the advancement of virtual reality (VR), researchers have developed remote virtual rehabilitation systems with sensors such as inertial measurement units. A functional garment with an integrated wearable sensor can also be used for real-time sensory feedback in VR-based therapeutic exercise and offers affordable remote rehabilitation to patients. Sensors integrated into wearable garments offer the potential for a quantitative range of motion measurements during VR rehabilitation. In this research, we developed and validated a carbon nanocomposite-coated knit fabric-based sensor worn on a compression sleeve that can be integrated with upper-extremity virtual rehabilitation systems. The sensor was created by coating a commercially available weft knitted fabric consisting of polyester, nylon, and elastane fibers. A thin carbon nanotube composite coating applied to the fibers makes the fabric electrically conductive and functions as a piezoresistive sensor. The nanocomposite sensor, which is soft to the touch and breathable, demonstrated high sensitivity to stretching deformations, with an average gauge factor of ~35 in the warp direction of the fabric sensor. Multiple tests are performed with a Kinarm end point robot to validate the sensor for repeatable response with a change in elbow joint angle. A task was also created in a VR environment and replicated by the Kinarm. The wearable sensor can measure the change in elbow angle with more than 90% accuracy while performing these tasks, and the sensor shows a proportional resistance change with varying joint angles while performing different exercises. The potential use of wearable sensors in at-home virtual therapy/exercise was demonstrated using a Meta Quest 2 VR system with a virtual exercise program to show the potential for at-home measurements.

## 1. Introduction

Virtual physical therapy, commonly called telerehabilitation, is a remote medical service that enables patients to access physical therapy sessions online or through other digital simulation channels. Virtual reality (VR) in physical therapy provides patients with an immersive experience that can improve their motivation, engagement, and participation in rehabilitation exercises. Prior research has demonstrated promising outcomes when combining VR and physical therapy for the upper and lower limbs and the performance of general tasks [1].

Pelaez-Velez et al. [2] used VR and video games in physical therapy. They found that treating stroke patients using VR in addition to a conventional physical therapy strategy resulted in considerable improvements in balance, gait, trunk control, and functional level of gait. Choi and co-workers [3] demonstrated that commercial gaming-based VR movement therapy was as successful as a traditional occupational treatment for recovering upper extremity gross motor function and ADL activities for daily living in subacute stroke patients with moderate-to-severe motor impairment. During and after the COVID-19 pandemic, there has been an increase in research studies that are focused on developing methods for at-home physical therapy with minimum or no visits to the clinic. In their study on the feasibility of VR exercise at home, Groenveld et al. [4] demonstrated that using VR for at-home physical and self-administered mental exercise is practical and well-received by roughly three-quarters of patients with post-COVID-19 conditions. Researchers have performed several studies [5,6,7] to investigate the use of VR in post-stroke upper extremity rehabilitation and show that VR-based rehabilitation is accepted among patients.

Feedback during exercise, such as angle movement or controlled movement, is an essential component of any physical therapy program, whether it is delivered in person or virtually. In virtual physical therapy, feedback can be provided in several ways, such as video conferencing, written and verbal feedback, and wearable technology. Motion sensors, biofeedback devices, VR headsets, and smart clothing are some examples of wearable technology that can be utilized to track patients’ progress. Human motion tracking and feedback can be visual or non-visual. Visual tracking uses body markers and camera-based monitoring. Non-visual tracking is based on inertia/magnetic-based sensors and other approaches [8]. Inertial Measurement Units (IMU) are frequently used sensors in human motion tracking. IMUs combine angular turning rates from gyroscopes and linear acceleration from accelerometers to form an integrated motion unit. IMUs were chosen for their portability and low cost and because they accurately simulate the user’s motion. Microsoft Kinect (Microsoft Corp, Redmond, WA, USA) is a standard low-cost sensor that can measure posture and balance during motion, though in their research, Yu et al. [9] found that Kinect shows poor performance in measurement when compared to a goniometer. 

Jovanov et al. [10] used a combination of motion sensors, SpO_2_, electrocardiogram (ECG), and tilt sensors to create a body area network to record data through a network coordinator device and transfer it via Bluetooth to the computer. Fergus et al. [11] developed a wireless body sensors network using motion sensors attached to different body parts and collected the acceleration data of these sensors wirelessly. Attaching the sensors to the body is uncomfortable to wear in this approach. Maskeliunas et al. [12] used depth sensors and machine learning for precise human posture and motion analysis in rehabilitation exercises. These sensors are small electronic devices attached to the human body to record feedback that makes the user uncomfortable. Alexandre et al. [13] developed a physio wear system where piezoresistive Flexi Force A201 and Flex Sensors 2.2 were embedded in the glove for each finger, and data were transmitted wirelessly through Bluetooth protocol. These smart gloves are easy and comfortable to wear; however, the challenge is integrating these (flexi force and Flex sensor) with fabric due to dissimilarity in the material of sensor and fabric. In the last decade, significant research has focused on developing smart garments. Several types of sensors can be used in smart garments, such as accelerometers, gyroscopes, heart rate monitors, temperature, pressure, ECG, stretch, and moisture sensors. Most of these sensors are metallic and need special processes to integrate seamlessly with fabric.

Carbon nanotubes possess exceptional mechanical, electrical, and thermal properties. Due to their high aspect ratio (length/diameter), they create an electrically conductive network at low concentrations. A network of carbon nanotubes can be arranged in a flexible and interconnected manner to create a coating on the textile, enabling piezoresistivity. The piezoresistive behavior of carbon nanocomposites has led to researchers’ development of multiple sensor applications in the last two decades. When mixed with a polymer, carbon nanotubes create an electrically conductive network at small concentrations [14] and resistance changes with strain [15]. Various polymer-based piezoresistive carbon nanotube composites are investigated as stretch sensors for human motion analysis [16,17,18,19,20,21]. The primary challenge with polymer-based sensors is their dissimilarity from the fabric’s physical properties, making it difficult to integrate with garments. Doshi et al. [22,23,24] processed functionalized carbon nanotubes through electrophoretic deposition on commercially available knitted fabric and developed a sensor with ultrahigh sensitivity for human motion analysis. These sensors show high sensitivity to flexion and extension when tested on an elbow or knee. 

In this work, we investigate a piezoresistive nanocomposite sensor created by dip-coating an everyday knit fabric used in compression garments in an aqueous dispersion of carbon nanotubes and polymer solids. The resulting fabric sensor is breathable and soft, can be sewn directly into a compression garment, and is highly sensitive to extensional strain/stretching deformation. The sensor was integrated into a compression sleeve and tested while positioning the sensor on the elbow joint. First, a calibration curve was created by measuring the elbow angle using a digital goniometer and recording resistance change. The sensor response was recorded during predefined path tracing using the Kinarm end point robot, and elbow angle change was extrapolated from the calibration curve. Different reaching tasks were developed with a custom VR environment and replicated by the Kinarm robot in a virtual setting. The carbon nanotube sensor located at the elbow on a compression sleeve is evaluated with Kinarm and VR exercises. In addition, a commercially available Meta Quest 2 VR system was used to perform VR video game-based stretching exercises, and the electrical resistance change in the sensor of the sleeve was evaluated with different types of elbow movements during exercise. 

## 2. Materials and Methods

In this research, carbon nanotubes are directly hybridized on commercial knit fabric. The fabric is tested in uniaxial elongation using a screw-driven mini tester (Instron 5848, Instron, Norwood, MA, USA). This sensor shows an ultrahigh response in flexion, as discussed in research by Doshi et al. [25]. The sensor is attached to the compression sleeve, and the sensor response, electrical resistance change with elbow angle change, is validated using the Kinarm robot. The sensor is also tested for VR-based tracking exercises. 

### 2.1. Carbon Nanocomposite Processing and Specimen Preparation

**Figure 1:**
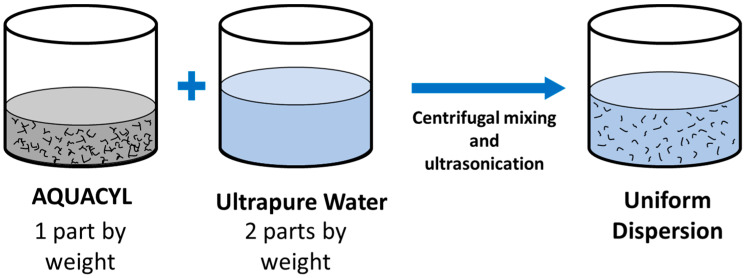
The process for preparing a uniform dispersion of carbon nanotubes for dip coating.

A dip-coating process was used to coat carbon nanocomposite on commercial knit fabric using commercially available multi-walled carbon nanotube aqueous dispersion with 3% loading of carbon nanotubes by weight (Aquacyl™, Nanocyl SA., Sambreville, Belgium). Two parts of ultrapure water are added to 1 part of Aquacyl by weight to lower the dispersion viscosity. Uniform dispersion is achieved by processing using a centrifugal mixture (THINKY^®^ ARM-310, THINKY, Laguna Hills, CA, USA) at 2000 RPM for 120 s and 30 min sonication in an ultrasonic bath (Branson^®^ 1510, Emerson Electric Co., St. Louis, MO, USA), Figure 1. A commercial knitted fabric used for compression garments is selected, which comprises nylon, polyester, and elastane. The nylon and polyester give excellent wear resistance, and elastane ensures high stretchability and resilience. To coat the fabric, sonicated dispersion is poured into a flat-bottomed glass container, and a piece of fabric is dipped for 10 min on each side. The coated fabric was dried in a convection oven for 30 min at 150 °C. 

**Figure 2:**
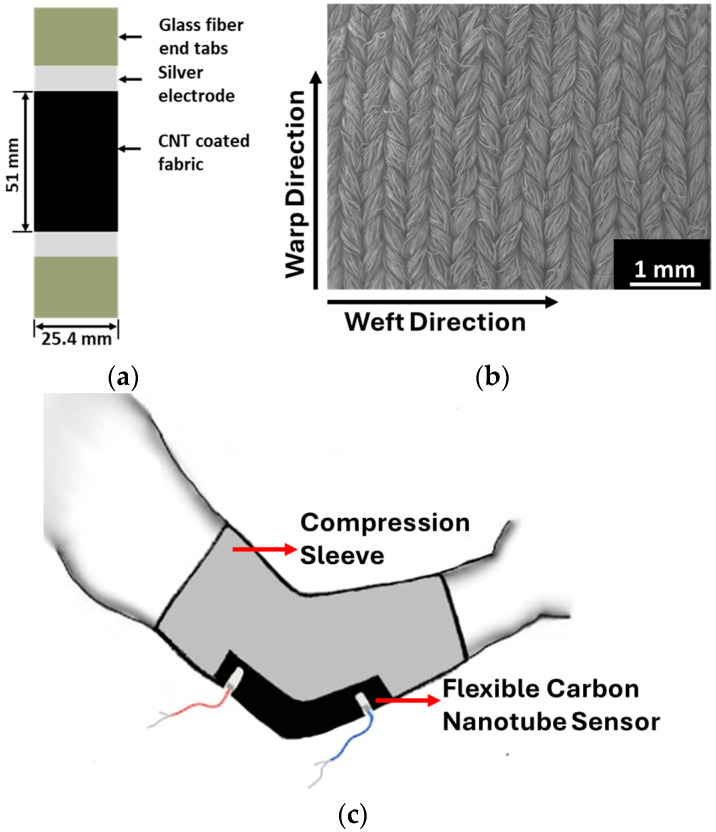
(**a**) Schematic of the specimen utilized for uniaxial testing in the warp direction; (**b**) scanning electron micrograph of weft knit fabric showing the looped structure and (**c**) schematic of a compression sleeve with a carbon nanotube sensor sewn onto the elbow location.

The specimens were prepared for the axial stretch testing by cutting carbon nanotube-coated fabric to 100 × 25.4 mm size. As shown in Figure 2a, electrodes were created 51 mm apart using conductive silver paint (Flash Dry, SPI Supplies, West Chester, PA, USA), and lead wires were then affixed to the electrodes using a two-part conductive silver epoxy resin (EPOXIES^®^ 40-3900, Epoxies, Etc., Cranston, RI, USA). Additionally, non-conductive glass fiber end tabs were attached to the ends of the specimen to ensure the straightness of the fabric and electrically isolate the sensor from the metallic grips of the testing machine. A scanning electron microscope (SEM) image of the knit fabric is shown in Figure 2b, revealing the looped structure and the warp and weft directions. It was demonstrated in our prior research that the warp direction of the fabric shows the highest sensing response [24]. The fabric specimens were tested in the warp direction under a controlled displacement rate of 0.05 mm/s.

To evaluate the response of the carbon nanotube-coated flexible sensor on the elbow, strips of the coated fabric measuring 130 × 38 mm were sewn onto a compression sleeve fabricated from a commercial knit fabric containing 82% nylon and 18% spandex. A zigzag sewing pattern is used to attach the sensor to the compression sleeve to avoid edge constraint, as shown in Figure 2c. Testing this sensor in a controlled environment is required to validate its response to the angle variation. Electrodes were applied to the sensor with a spacing of 101 mm by applying silver paint and attaching electrically conductive wires using 2-part silver epoxy. Before testing with the Kinarm end point robot and the VR environments, a calibration curve was generated for the participant wearing the compression sleeve along with manual measurements. The compression sleeve is worn on the arm, and the arm is flexed from an entirely straight position in a sequence of steps. A digital goniometer was used to measure the elbow angle, and the electrical resistance of the sensor was recorded during flexion to establish a calibration curve. A voltage divider circuit was used to record the sensor response using a constant voltage of 5 V throughout all tasks. The sleeve signal and position of the handle of the Kinarm are recorded in real time at a frequency of 1000 Hz. 

### 2.2. Testing Protocol for Validation with the Kinarm

We used an end point robot (Kinarm Endpoint Lab, BKIN Technologies, ON, USA), which consisted of a robotic arm that allows hand motion in a 2-D horizontal plane. The robotic arm has a handle located at the end, which participants grasp with their dominant hand. The resistance of the sensor varied with changes in elbow angle. Each task was developed to capture different movements of the hand. Participants held the handle during the experiment, and the Kinarm robot drove the handle along the assigned path. The participant sits, keeping their back straight, and supports their forehead at a designated point in the Kinarm setup, Figure S1. The participant applied no force/resistance to handle movement during the tasks. A minimum jerk trajectory was used to move the handle from a start position to an end position on the trajectory. The following four different tasks were created on the Kinarm robot.

**Figure 3:**
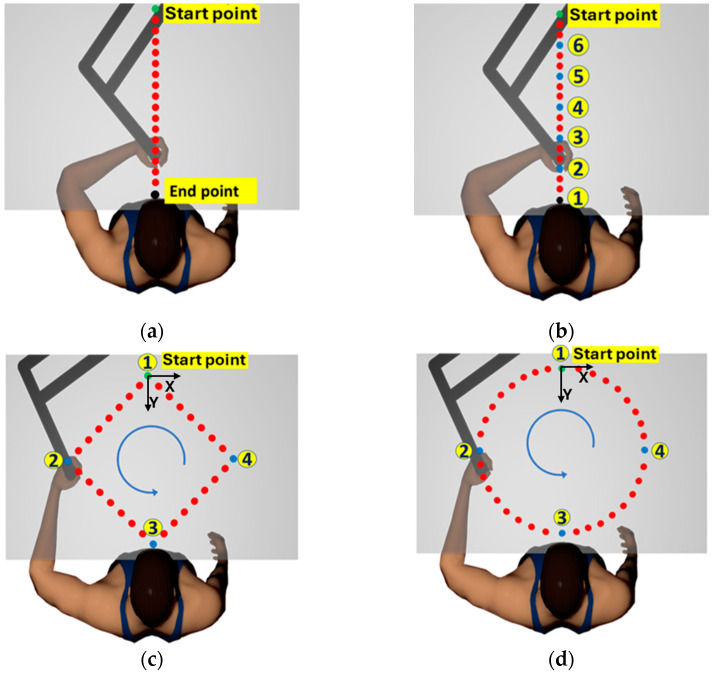
Sketches showing arm movements used for testing (**a**) Constant amplitude straight line movement task for a displacement of 30 cm in the vertical direction; (**b**) variable amplitude straight line movement task from 30 cm to 5 cm when the handle moves from the start point to points 1-6, where the amplitude decreases by 5 cm between each point; (**c**) in-plane two-dimensional movement of the arm in diamond path with angle measured at four end points; and (**d**) in the plane two-dimensional movement of the arm in a circular path with an angle measured at four end points. The arrow indicates the direction of movement.

*A constant displacement straight-line motion task* is created to validate the sensor repeatability under constant amplitude movement of the arm. As shown in Figure 3a, in this task, the arm handle is moved from the start point to the end point, separated by 300 mm. The change in elbow angle is measured for this motion using a digital goniometer.

*A variable displacement straight-line motion task* is created to validate the sensor’s response with the variation in the elbow angle. As shown in Figure 3b, the starting point was at a forward distance of 300 mm from the participant’s position. Different endpoints, 1–6 were used at distances that increased in steps of 50 mm from the start point (indicated as blue dots). The maximum distance traveled from the start point to point 1 in a straight-line motion is 300 mm and then decreases by 50 mm in each cycle, as shown in Figure 3b. Five cycles were repeated for each amplitude, and a change in elbow angle was measured at each repetition, i.e., start point and points 1 to 6, using a digital goniometer. 

*Two-dimensional movement in a diamond-shaped path* is created to capture the movement in two dimensions. Four in-plane points are created 1 (0, 0), 2 (−150, 150), 3 (0, 300), and 4 (150, 150), as shown in Figure 3c. The objective is to keep a consistent movement of the hand in the Y-direction. The robot was programmed to follow a straight-line path between points (1, 2, 3, and 4) starting from point 1 in the anti-clockwise direction. The elbow angle was manually measured at these four points. While tracing the diamond path, the handle is stopped at points 2, 3, and 4 for 250 ms. 

*Two-dimensional movement in a circular path* is created such that the circle passes through the same 4 points created for the diamond path. However, the robot is programmed to follow the circular part, starting from point 1 and passing through points 2, 3, and 4 while completing the cycle, as shown in Figure 3d. The handle moves continuously without any holds at intermediate points. 

### 2.3. Testing with Virtual Reality Configurations 

**Figure 4:**
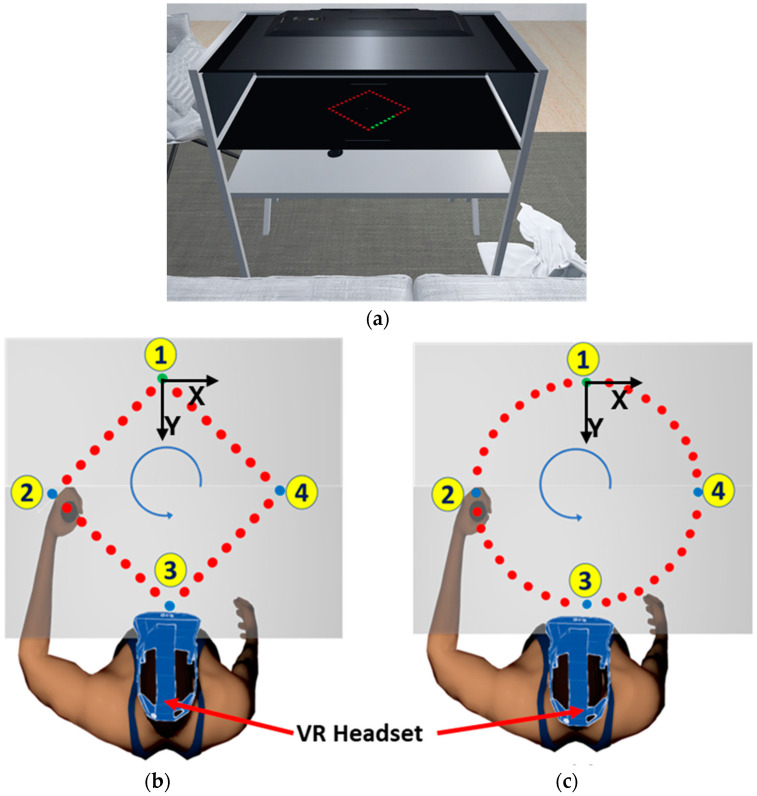
(**a**) Virtual model of the Kinarm in a home family room setting created in VR environment, (**b**) in-plane two-dimensional movement of the hand following a diamond path, and (**c**) in-plane movement of the hand following a circular path. The user moves the handle from point 1 to point 2, 3, and 4, following straight line and circular paths.

The overarching goal of the carbon nanotube sensor is to provide feedback and assessment for upper extremity rehabilitation, such as a change in the elbow angles while performing the exercise remotely controlled by a VR environment. The sensors are tested with therapeutic tasks to assess their effectiveness and usability in the VR environment. Unity game engine (version 2021.3.10f1) was used to create the virtual environment for upper extremity rehabilitation. We used the virtual reality toolkit (VRTK) for fundamental VR interactions and teleportation. The VR environment was developed to provide compatibility with various VR headsets by using the Unity OpenXR package. We used the HTC Vive Pro Eye VR headset, controllers, and tracking components in this test in conjunction with the end point robot and elbow sensor, Figure S2. The 3D models from Sketchfab were modified and added to the virtual setting, as shown in Figure 4a. 

The goal is to provide users with an engaging environment with feedback. A virtual model for the Kinarm robot was designed to mimic the Kinarm in a virtual environment. An HTC Vive tracker was attached to the mechanically controlled robotic arm to ensure movement in the two-dimensional plane. The participant wears the headset and sits in a straight position. The participant moves the robotic handle, and the movement of the virtual Kinarm is simulated in the virtual reality at the same time. 

As shown in Figure 4b,c, two tasks are designed for the upper extremity exercise, diamond and circle, like the Kinarm tasks. The testing protocol follows Kinarm testing. However, the participant uses a VR headset and follows the paths, diamonds, and circles created in a virtual environment. This is to create a similar task as performed with a position controlled Kinarm robot. The participant can follow the path at a speed comfortable to them. The change in elbow angle is recorded at the intermediate points. Tasks are performed while maintaining the in-plane positioning of the hand. The position data of the hand in the x–y plane is recorded simultaneously with the sleeve signal. In order to validate the concept for at-home virtual rehabilitation, a Meta Quest 2 and a commercial VR exercise game were used to demonstrate the ability to track stretching exercises, as a demonstration of range-of-motion tracking.

## 3. Results and Discussion

**Figure 5:**
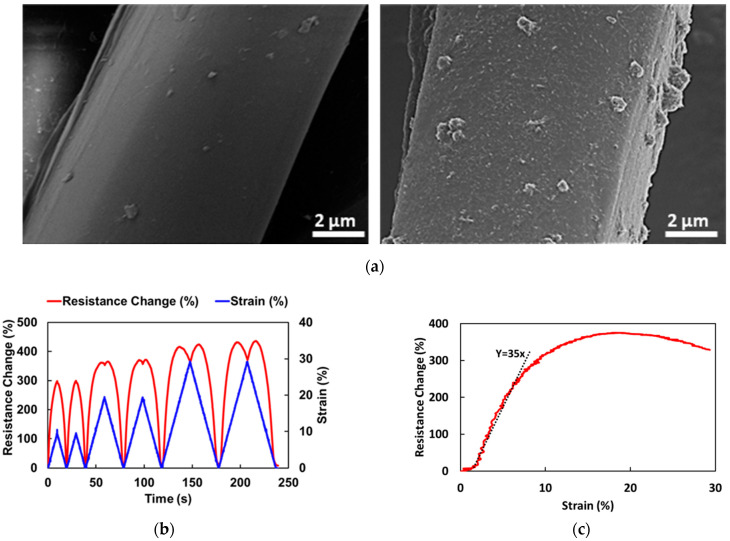
(**a**) Surface morphology of a fiber in a knit fabric before and after the carbon nanocomposite coating; (**b**) sensor response when tested for the uniaxial stretch in the warp direction for 10%, 20%, and 30% strain levels; and (**c**) resistance change against strain with a gauge factor of ~35 at low strains.

Characterization of the fabric sensor in tension was utilized to down-select different knitted fabrics in order to select materials for the elbow sensors. Figure 5a shows SEM micrographs of fibers in the knitted fabric before and after coating. The uncoated fibers show a smooth surface, and the coated fibers show a uniform carbon nanotube composite coating. The texture on the surface of the fibers is from the carbon nanotubes embedded in the polymer after drying. The nanotube dispersion/coating was also characterized using thermogravimetric analysis (TGA) and energy-dispersive X-ray spectroscopy (EDS). As per the technical data sheet, the dispersion is water-based, contains some surfactants, and has 3% multi-walled carbon nanotube loading. TGA analysis shows that the dispersion contains 94–95% water, and 5–6% is carbon nanotubes and surfactants, Figure S3. The EDS analysis reveals the elemental composition, with the majority being carbon and oxygen, Figure S4a,b. There is also a presence of 2.7 wt% sodium, likely because of the surfactants used for dispersion, Figure S4c. Additional details about the nanocomposite coating on the fabric and sensor response can be found in references [23,24,25].

Specimens from candidate fabrics were stretched uniaxially in the warp direction. Figure 5b shows the resistance response of the sensor selected for the elbow sensor at progressively increasing cyclic loading. The sensor’s resistance–strain response of the sensor is nonlinear and increases up to about 20% strain before a decrease in resistance at higher strain, more than 20%. With each cycle, the sensing response is repeatable. Figure 5c shows the resistance–strain behavior up to 30% strain. The sensing response is linear up to approximately 5% strain. The sensor sensitivity in the linear range is described by the gauge factor or the slope of the resistance change-strain. For this fabric, the initial gauge factor is 35. For comparison, a traditional metallic strain gauge has a gauge factor of approximately 2. At 10% strain, the resistance change is close to 300%. The nonlinearity of the resistance response is associated with the nonlinear behavior of the knit fabric. As shown in Figure 2b, yarn travels in a weft direction and forms loops in a typical weft knit fabric. On stretching in the warp direction, these loops become elongated in the warp direction, and the resistance increases drastically, even at low strain. After this initial stretching, the resistance continues to increase due to straining of the fibers. The decrease in resistance at higher strains, in the 20–30% range, is due to the contraction of the fabric in the transverse direction. This causes the loops in the fabric to contact each other, creating new conducting pathways, as discussed in [24]. 

Our earlier research [24] has demonstrated the sensor response in uniaxial stretching and sensitivity in measuring joint motion. It was established that bi-axial stretching plays an important role in the sensor response. The skin around the elbow joint has been observed to extend 35–40% in length and 15–22% in circumference [26]. SEM micrographs in Figure S5a show the microstructure of an unstretched knit fabric and Figure S5b shows a fabric stretched biaxially, where the loops are elongated and separated. When the sensor is integrated into an elbow compression sleeve, the sensing range is increased because of the constraint of the arm, resulting in biaxial stretching. This constraint keeps the loops in the fabric from contacting at higher amounts of extension. As a result of the biaxial stretching and the different sensor deformation mechanisms compared to uniaxial testing, the sensor must be calibrated while integrated into the compression sleeve. When used on the elbow, the monotonicity of the resistance response is maintained for complete elbow joint angle movement, as shown in Figure 2c of [24].

**Figure 6:**
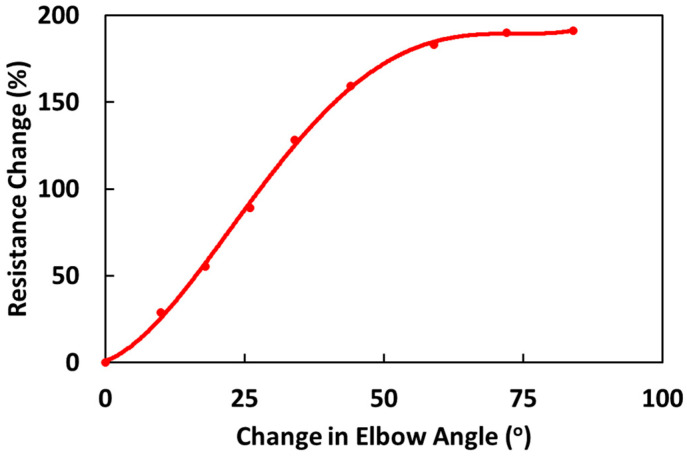
The calibration curve generated with the participant with a compression sleeve on the arm for increasing change in elbow flexion angle.

The sensor does need to be worn over the elbow, but since the response is primarily due to the local stretching right at the tip of the elbow, slight misalignment has less of an overall effect. The calibration curve will vary from person to person due to variability in arm dimensions/ elbow movement. We need to create a calibration curve for every individual who is using the sensor. Sensing response, and hence the calibration curve, also depends on the knitted structure of the fabric, carbon nanotube composite processing parameters, sleeve material composition, and the sleeve’s fitting. To generate the calibration curve, the percentage resistance change is plotted with the change in elbow angle measured using a goniometer. Figure 6 shows the sensing response and the calibration curve fitted with a fourth-order polynomial. This calibration curve can then determine flexion without using a goniometer. The graph shows that the response is linear up to an angle of 50° flexion with a linear fit to a correlation of 0.996. Although the curve flattens at higher elbow angles, the resistance response in this range of angles is always increasing. The resistance response over the full range of motion is large, over 180%. It is noted that, due to the strain in the knitted fabric caused by the subject wearing the sensor with their arm fully extended, the initial baseline resistance is 40% higher than the fabric in the fully relaxed state. This is because the sensor experiences stretching in both the weft and warp direction when the sleeve is worn on the arm.

**Figure 7:**
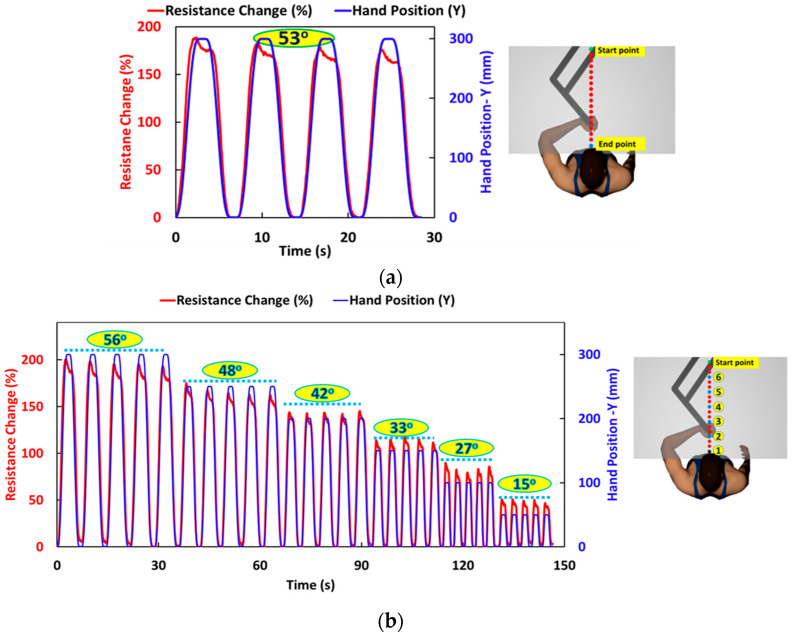
(**a**) Resistance change (%) in the arm flexion from moving start point to end point with a total change in angle of 53°, showing repeatability of the sensor response and (**b**) resistance response of sleeve in variable amplitude straight line motion with change in elbow angle when handle moves from start point to points 1–6, starting from an amplitude of 30 cm and decreasing by 5 cm for each subsequent point.

The participant performs a constant displacement straight-line motion task by holding the handle with the left hand. Here, we define the positive *Y*-axis as towards the participant, considering the start point as the origin. The sensor’s electrical resistance change is plotted in real-time with the hand position in the Y-direction, as shown in Figure 7a. The elbow angle change between the start and end points is 53°, and the response is repeated for multiple cycles. For this measurement, the arm is partially flexed at the start point of the motion by the Kinarm, so there is some initial resistance change due to this partial flexure. Multiple cycles were performed to examine the response over time. For each extension/flexion cycle, there is a slight decrease in the sensor resistance in the flexed position, which is likely due to slight sliding of the sensor over the elbow during the hold increment. The resistance returns to the initial measured resistance on each cycle when extended. 

Figure 7b shows the results of the straight-line motion task with variable amplitude. In each position, the measured resistance tracks directly with the hand position for each amplitude. The angle change measured at each hand position tracks closely with the calibrated sensor response on the subject’s arm. The figure shows five cycles for each hand position, and a consistent sensor response is observed for each amplitude. The average percentage change in resistance is given in Table S1. This shows the sensor response is repeatable with a small coefficient of variance (0.01–0.03) for a similar elbow angle change, and the response changes with the change in elbow angle.

*Two-dimensional tracing in the diamond and circular path* tasks are created to capture the in-plane motion of the hand. For both two-dimensional in-plane motions, the start point is considered as the origin with a positive Y-axis towards the participant from the start point 1 and a positive X-axis from left to right in the plane. The motion is recorded in the X–Y plane at every data point. The resistance change in the diamond path tracing was close to 170% for the hand movement from start point 1 to 3, a total elbow angle change of approximately 53°. This change was close to 180% for a 55° elbow angle change for circular path tracing.

**Figure 8:**
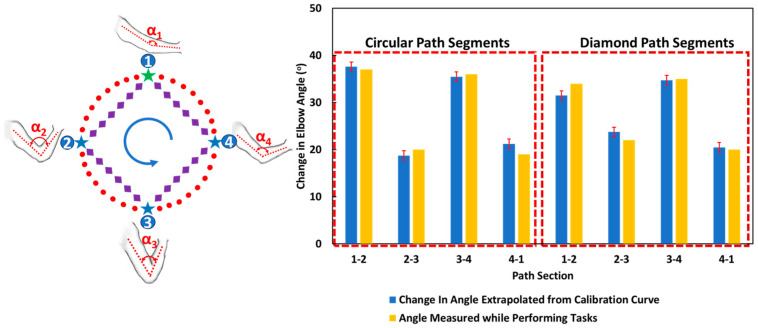
Elbow angle change was calculated using a goniometer during activity for points 1, 2, 3, and 4, and the change in angle was extrapolated from the calibration curve using resistance change values for the different sections of circular and diamond paths between points 1, 2, 3, and 4. Dotted boxes separates the data for circular and diamond path segments.

The percentage resistance change is calculated throughout the path tracing for the multiple-cycle experiment. Resistance changes for sections 1–2, 2–3, 3–4 and 4–1 is calculated. A change in angle is calculated for the corresponding resistance change values from the calibration curve. Figure 8 compares angles calculated manually using a goniometer during exercise in the first cycle and angles extrapolated from the calibration curve using resistance change values for the different sections of circular and diamond paths for multiple test cycles. The angle measured are comparable and within 90% accuracy of the angle measured manually during the task with a very small coefficient of variation (0.02–0.070), as shown in Table S2. The error bars represent the variation of motion under many cycles and may be because of slight changes in the user’s posture. The sensor shows good repeatability for the cycles tested and can reflect the elbow angle change based on the in-plane motion of the handle. In addition, the sensor is tested for 1000 cycles in uniaxial tension and shows a repeatable response, as shown in Figure S6a,b.

**Figure 9:**
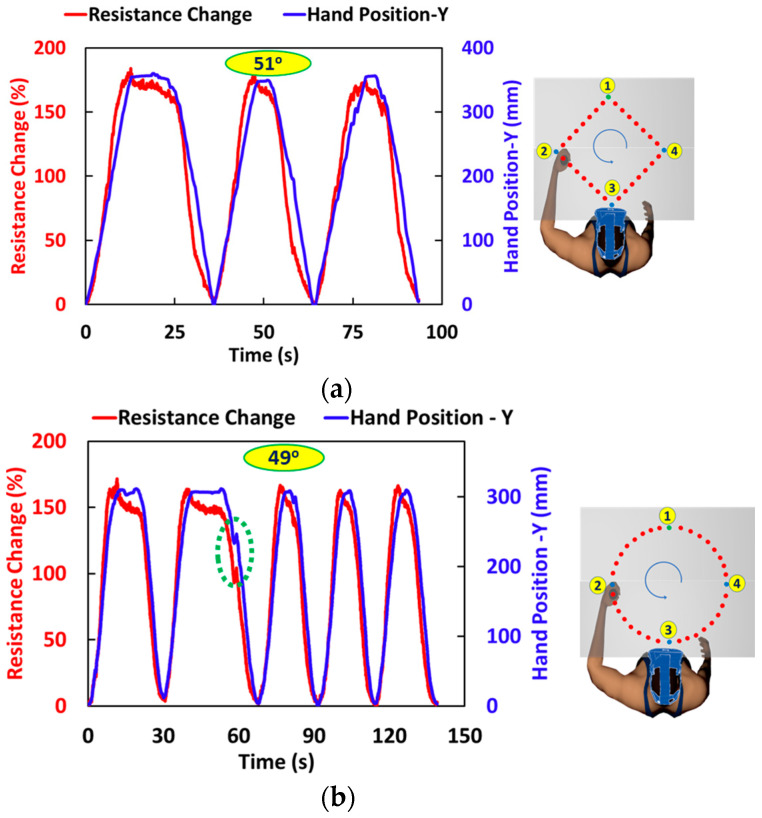
Response of the sensor in compression sleeve during elbow motion with virtual reality task (**a**) diamond path tracing, and (**b**) circular path tracing with a deviation from the intended path (dashed area of the second cycle). The user moves handles from point 1 to points 2, 3, and 4, following a straight (for diamond path) or circular path. A dotted encircled point is a mistake made by the participant while performing a circular task.

As explained in the experimental section, two similar paths are traced in the VR environment task, as with Kinarm. Unlike the Kinarm experiments, the time to complete the task is up to the participant. In both tasks, a change in elbow angle is measured at each position. The resistance change is recorded and plotted against the Y-displacement position of the hand. In X-direction hand movement, the change in the angle of the elbow is not significant. The result of the VR task is like the mechanically controlled hand movement. In diamond-shaped hand tracing, the resistance change is close to 180% with an angle change of approximately 51°, as shown in Figure 9a. The time to complete the task is different for each cycle; however, no significant difference is observed in the resistance change in the sensor for the same angle change in the elbow.

The resistance change is in the same range because the angle of the elbow is in the same range. Also, there is no hold time for any intermediate position. Therefore, the resistance curve is continuous. For circular path tracing, the change in resistance is lower than the mechanically controlled one. The difference is because of the different changes in angle. This difference in the angle is because of the uncontrolled movement of the hand based on human judgment. During the hand movement, the participant made a mistake following the path, which is captured by the sensor, as shown in Figure 9b. 

**Figure 10:**
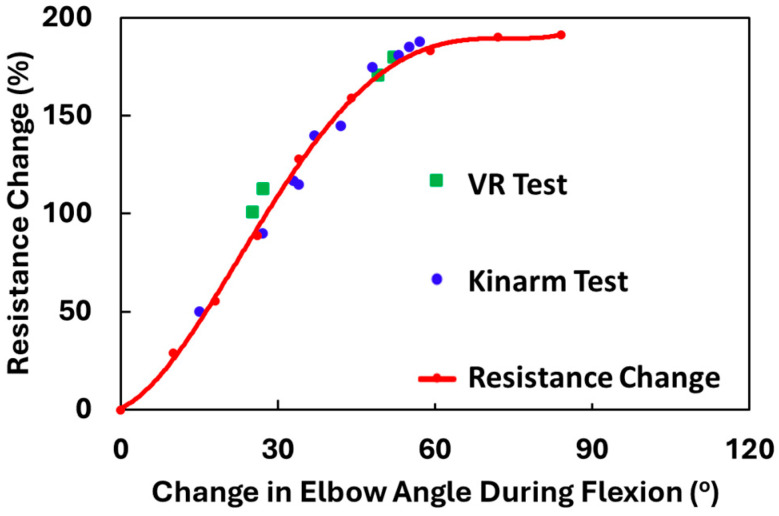
Calibration curve with resistance change plotted against change in elbow angle during flexion, while performing the tasks on Kinarm end point robot and VR.

The sensor response is repeatable under multiple tests and varying elbow angles. To minimize sensor-to-sensor variation, we control the manufacturing parameters such as the coating time, the dispersion concentration, and the areal weight of the carbon nanocomposite deposition. We ensure the baseline resistance of the sensors is in a similar range and then, while taking the measurements, always normalize the change in resistance with baseline resistance. However, responses also vary depending on factors such as the subject’s arm size and the positioning of the sensor on the arm. As a result, it is essential to calibrate the sensor for each person while wearing the sensor. The calibration curve is generated based on the angle and sensor response measured for arm flexion. Figure 10 shows the resistance response measured at varying angles during both Kinarm and VR tests. When all the data, resistance changes with change in elbow angle, generated in the Kinarm and VR tests, is plotted, the data points lie close to the calibration curve. This shows that the calibration curve can enable the measurement of the change in elbow angle based on the resistance change during exercise, where the position is not dictated by either the Kinarm or VR systems. 

## 4. VR Application in Home Exercise

The rehabilitation robot is an expensive research tool that would not be used in an at-home environment. In this research, the experimental methodology was first to use the robot to control the motion and validate the range of motion of the sensor. The robot was then replicated in a VR environment, where the user controls the path, but the robot also monitors the path, and the sensor response is recorded. Since optical tracking of the VR gives little information on the range of motion, the VR system combined with the fabric-based sensor offers the potential for at-home exercise monitoring and providing data on range of motion.

**Figure 11:**
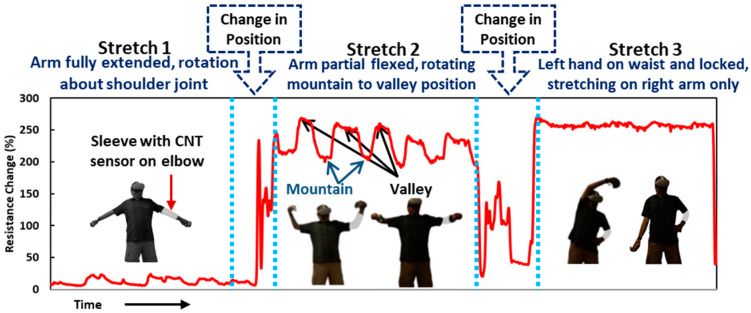
Participant testing with commercial stretching exercise available on Oculus Quest with CNT sensor on the sleeve in one hand and sleeve response, percentage resistance change, for the three different types of stretch exercises. Mountains and valleys in the resistance curve and the corresponding arm positions during stretch 2 are shown with arrows.

To demonstrate the potential applicability of the sensor in tracking VR exercise, the carbon nanotube flexible sensor integrated with a sleeve is used to get the sensing response while performing a virtual reality stretching exercise on Meta Quest 2. A commercially available exercise video game was selected to perform with the fabric sensor sleeve, and the response was recorded. The data were recorded using a voltage divider circuit and an inexpensive miniaturized data logging system (Arduino Nano) suitable for potential home use. The motions were recorded on video using a camera located at a single position. Multiple stretching tasks are performed during the exercise. First, three stretching task data are shown in Figure 11. Three different stretching exercises are performed, and a five-point average resistance change is plotted for the task’s duration. Each task response is explained below:

*Stretch 1*—In the first stretch task, the arms are kept straight and abducted about the shoulder joint. In this task, there is minimal/no change in the elbow angle, so there is an insignificant change in electrical resistance. The small resistance change corresponds to each cycle rotation and is primarily due to slight stretching of the fabric while rotating the arm about the shoulder. 

*Stretch 2*—In the second stretch task, the arms are flexed (close to 90°), and rotated to the position from mountain to valley, as shown in Figure 11. Due to flexion, the sensor’s resistance increases by approximately 250%, and a decrease of ~50% in resistance is noticed while hands are brought to the valley position. In this task, visually, there is no noticeable change in the elbow angle, but there is an actual change in the elbow angle captured by the sensor.

*Stretch 3*—In the third stretch task, the hand with the carbon nanotube sensor on the elbow is kept on the waist. The arm is flexed and kept in a fixed position, and the exercise is performed keeping the hand static. There is no change in elbow angle in this task due to the fixed position, so there is no resistance change in the sensor during the task. In an earlier experiment, an end point robot was used to ensure controlled, repeatable movement of the arm; however, when using the sensor with Meta Quest 2, arm movement is not restricted in any plane. The response is dependent on the elbow angle change, irrespective of the hand position. Multiple sensors can capture the hand’s overall motion.

## 5. Conclusions and Future Work

Exercises as part of physical therapy are often critical to achieving full recovery from injury. However, a large number of patients often do not adhere to the exercise regimens, slowing recovery. Virtual reality exercises offer the potential for a game-like immersion where exercises can be performed. Optical tracking in VR often does not accurately track the range of motion of specific movements. This research has investigated a wearable sensor that can be fully integrated into a garment for the measurement of joint angle rotation. These sensors integrated into wearable garments offer the potential for a quantitative range of motion measurements during VR rehabilitation. In the long term, VR and wearable sensors can be used to provide direct feedback on home exercise to both the patient and clinician during rehabilitation. A commercially available weft-knitted fabric composed of polyester, nylon, and elastane, was used to create an elongation sensor by applying a fiber-level nanocomposite coating. The carbon nanotube-based nanocomposite is piezoresistive, where the electrical resistance changes with applied strain. Axial extension tests were utilized to down-select the fabric for sensor integration into the garment. The resulting sensor is breathable and soft to the touch and shows high sensitivity to axial elongation in the warp direction of the fabric, with an average gauge factor of 35. 

In order to validate these sensors for use in a wearable garment, the sensor was integrated into a compression elbow sleeve. Measurements were taken using a Kinarm end point robot as well as a VR environment to validate the sensor response and repeatability of the sensor to detect changes in joint angle. Compression sleeve sensor measurements were taken with the authors wearing the sensor garment. The VR environment was designed to replicate the robotic motion. During the tests, the elbow joint angle was also measured with a digital goniometer. The sensor shows transverse sensitivity in deformation, and because it is integrated into a compression sleeve, the transverse constraint due to the user’s arm alters the sensor response. A calibration protocol was developed to establish the sensor response to joint rotation. The calibration curve was fitted with a fourth-order polynomial. The sensor was then validated using a Kinarm end point robot and a VR environment to measure the range of joint motion. Specific tasks, including uniaxial extension along with diamond and circular hand motion patterns were designed to simulate an exercise. Sensor data were acquired in real-time with the Kinarm and VR motion and the elbow joint angle was measured using a digital goniometer. The measured joint angle in both the Kinarm robot and the VR environments showed that the calibration curve generated was highly accurate. 

The wearable sensor can measure the change in elbow angle with more than 90% accuracy while performing these tasks, and the sensor shows a proportional resistance change with varying joint angles while performing different exercises. Tables S1 and S2 show the coefficients of variation for in-line motion and two-dimensional motion in the range of 0.01–0.04 and 0.02–0.07, respectively. The maximum difference in angle extrapolated from the sensor’s resistance change using a calibration curve and angle measured using a digital goniometer while performing the task is 3°. The potential use of wearable sensors in at-home virtual therapy/exercise was demonstrated using a Meta Quest 2 VR system with a virtual exercise program to show the potential for at-home measurements. For the robotic and VR measurement, the tasks were constrained to a 2-D plane. In order to demonstrate the potential for VR exercise combined with an accurate range of motion measurements for home use, a low-cost data logging system was used to acquire movement data utilizing a Meta Quest 2 VR system and a VR exercise game. The tasks performed demonstrate the potential for use as an at-home exercise system that can potentially track and log a quantitative range of motion data. The calibration for joint rotation depends on the user. We have not conducted any research concerning the hygrothermal effect on this sensor. However, based on earlier investigations on similar nanotube-based sensors [27], the influence of temperature/humidity will be negligible compared to sensor response. Future work will evaluate the key sensing mechanism of the sensor and the response of the sensor under different parameters, such as fabric microstructure, the arm circumference of the user, and variation in speed of flexion and extension. With the integration of additional sensors in a compression garment in the shoulder area, the potential exists to capture a complete motion response. 

## Data Availability

The datasets presented in this article are not readily available because the data are part of a future study. Requests to access the datasets should be directed to the corresponding author.

## Supplementary Materials

The following supporting information can be downloaded at: https://www.mdpi.com/article/10.3390/s24154894/s1, Figure S1: Photograph of the participant gripping the Kinarm end point robot handle wearing the compression sleeve on their arm with the integrated carbon nanotube sensor located at the elbow joint. In this scenario, the robot drives the motion of the hand, and the sensor continuously records the flexion of the elbow. In the first cycle the elbow angle was manually measured using a digital goniometer at the four designated points in the pattern. Figure S2: Photograph of the participant wearing the VR headset, and gripping the Kinarm end point robot handle while wearing the compression sleeve on their arm with the integrated carbon nanotube sensor located at the elbow joint. The VR hand controller is attached directly to the Kinarm end point robot handle for optical tracking of the hand motion. In this scenario, the participant controls the motion of the handle while tracing a path programmed in the VR headset. The robot also monitors the hand position in the 2-D plane and the sensor monitors the flexion of the elbow. Figure S3: TGA thermograph of Aquacyl™ in an inert environment shows the effect of heat degradation including carbonization. TGA is performed using Netzsch TG 209 F1 Libra. In Aquacyl, water content is close to 95%, ~3% carbon nanotube loading, and approximately 2–2.5% is surfactant. Figure S4: EDS is performed using Auriga 60 cross beam with exciting voltage of 20 kV, (a) SEM micrograph of the carbon nanocomposite coated fabric representing the area for EDS analysis, (b) elemental composition primarily containing carbon and oxygen, 2.7% of sodium and small amounts (<1%) of calcium and aluminum and, (c) distribution of elements showing higher density of sodium is in nanocomposite coating rich area, likely due to the presence of sodium-based surfactants used in the dispersion. Figure S5: SEM micrograph of carbon nanocomposite coated knit-fabric (a) unstretched and (b) bi-directional stretched Figure S6: (a) Resistance change (%) in each cycle, in the first ten and last ten cycles in a 1000-cycle uniaxial tension test, showing no change in the overall sensor response, and (b) resistance change (%) with time for the first cycle and 991st cycle overlaid on the same time scale, showing no change in the individual cyclic sensor response. Table S1: Resistance change during variable displacement straight line motion task, while the user is holding the Kinarm end point robot handle and motion is programmed. Table S2: Change in angle extrapolated from the resistance value measured during two-dimensional movement in diamond and circular paths for the sections 1–2, 2–3, 3–4 and 4–1.
