# Supplementary material for: Characterizing the Sensing Response of Carbon Nanocomposite-Based Wearable Sensors on Elbow Joint Using an End Point Robot and Virtual Reality
Source: Sensors (Basel). 2024 Jul 28;24(15):4894. doi: 10.3390/s24154894 (PMC11314941; doi:10.3390/s24154894)
Supplement: Supplementary file 1 [file sensors-24-04894-s001.zip › sensors-3083487-supplementary.pdf]

Characterizing the Sensing Response of Carbon Nanocomposite-Based Wearable Sensors on Elbow Joint Using an End Point Robot and Virtual Reality

Amit Chaudhari [1], Rakshith Lokesh [2], Vuthea Chheang [3], Sagar M. Doshi [1], Roghayeh Leila Barmaki [3], Joshua G. A. Cashaback [2] and Erik T. Thostenson [4,*]

[1] Center for Composite Materials, University of Delaware, Newark, DE 19716, USA; amitc@udel.edu (A.C.); smdoshi@udel.edu (S.M.D.)
[2] Department of Biomedical Engineering, University of Delaware, Newark, DE 19716, USA; lokerak@udel.edu (R.L.); joshcash@udel.edu (J.G.A.C.)
[3] Department of Computer and Information Sciences, University of Delaware, Newark, DE 19716, USA; vuthea@udel.edu (V.C.); rlb@udel.edu (R.L.B.)
[4] Department of Mechanical Engineering, Department of Materials Science and Engineering, and Center for Composite Materials, University of Delaware, Newark, DE 19716, USA
* Correspondence: thosten@udel.edu (E.T.T.)

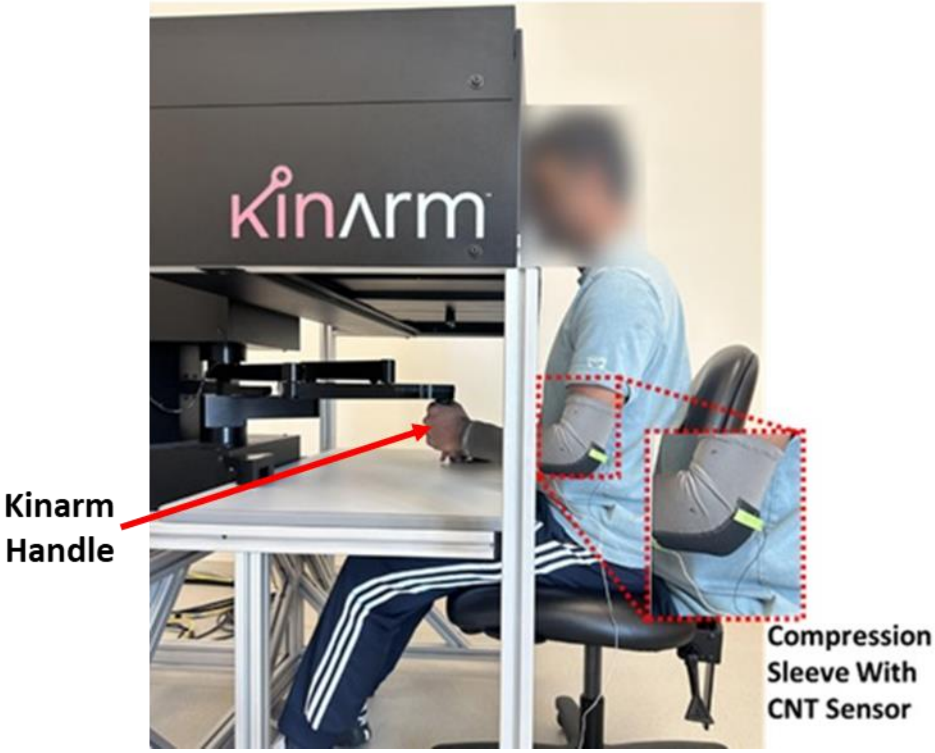

Figure S1: Photograph of the participant gripping the Kinarm end point robot handle wearing the compression sleeve on their arm with the integrated carbon nanotube sensor located at the elbow joint. In this scenario, the robot drives the motion of the hand, and the sensor continuously records the flexion of the elbow. In the first cycle the elbow angle was manually measured using a digital goniometer at the four designated points in the pattern.

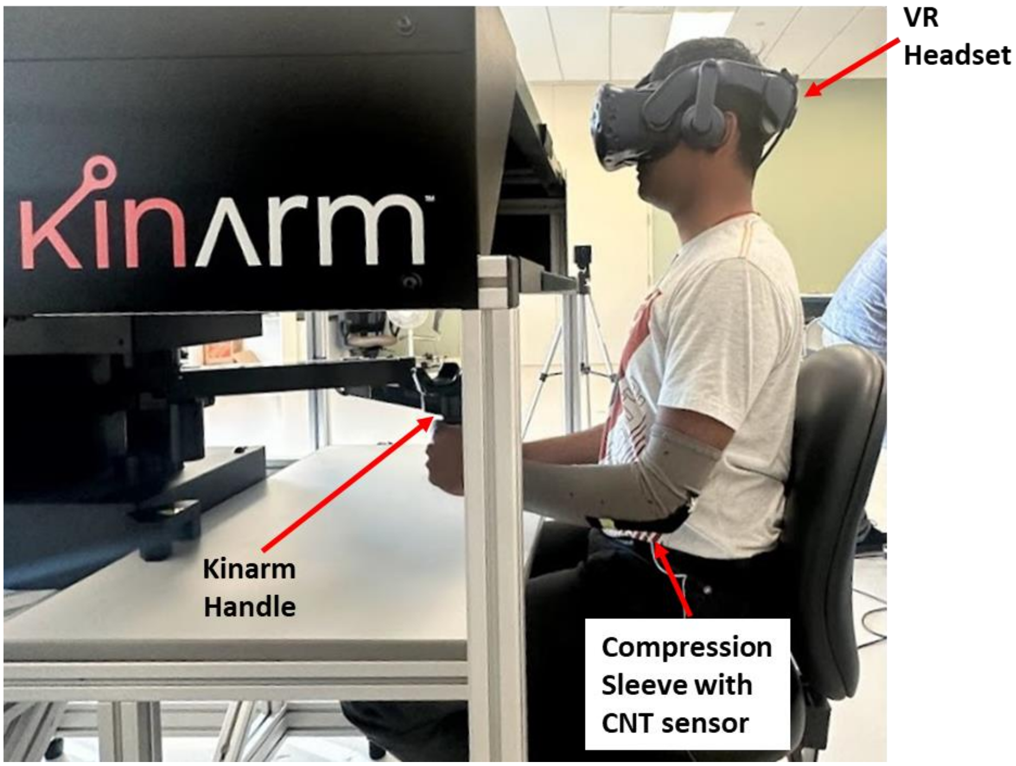

Figure S2: Photograph of the participant wearing the VR headset, and gripping the Kinarm end point robot handle while wearing the compression sleeve on their arm with the integrated carbon nanotube sensor located at the elbow joint. The VR hand controller is attached directly to the Kinarm end point robot handle for optical tracking of the hand motion. In this scenario, the participant controls the motion of the handle while tracing a path programmed in the VR headset. The robot also monitors the hand position in the 2-D plane and the sensor monitors the flexion of the elbow.

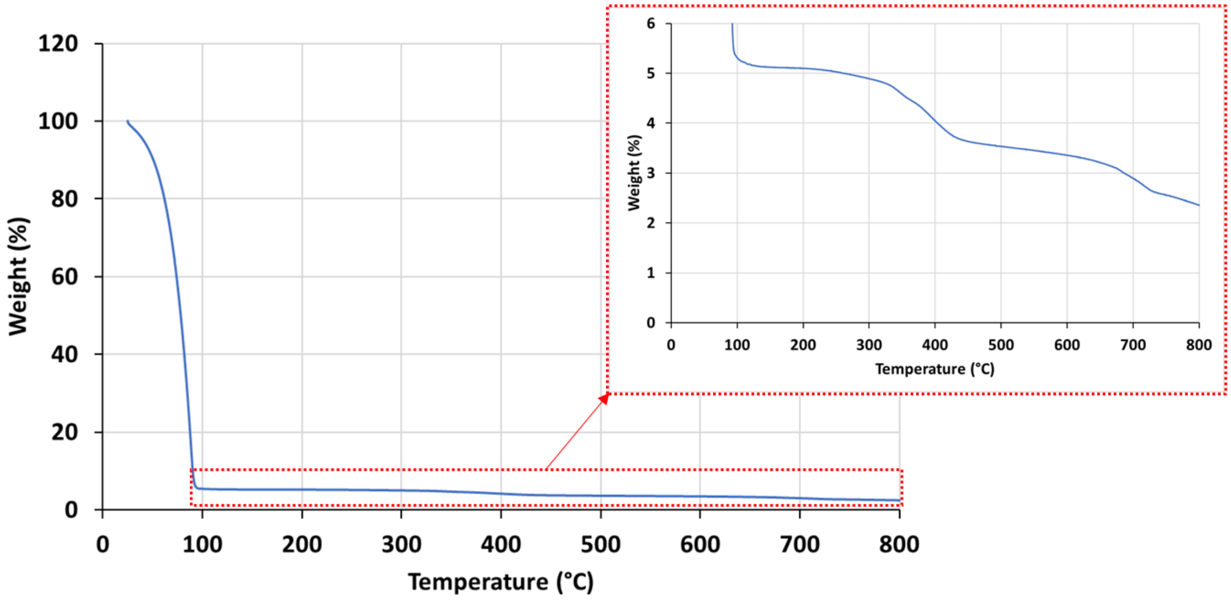

Figure S3: TGA thermograph of Aquacyl™ in an inert environment shows the effect of heat degradation including carbonization. TGA is performed using Netzsch TG 209 F1 Libra. In Aquacyl, water content is close to 95 %, ~ 3% carbon nanotube loading, and approximately 2-2.5 % is surfactant.

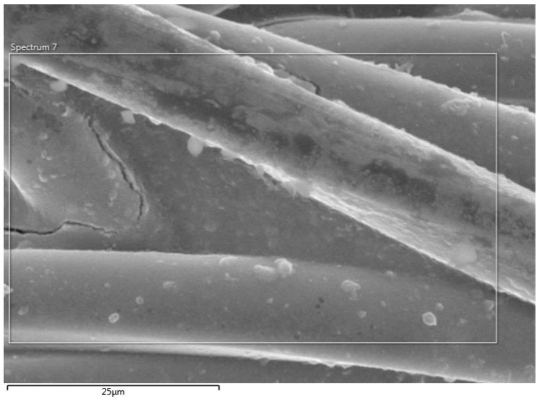

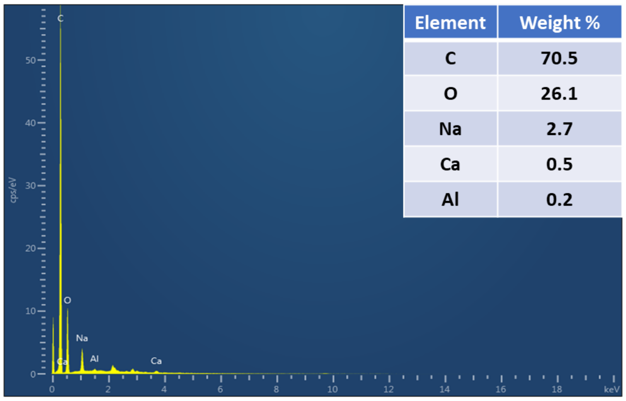

(a) (b)

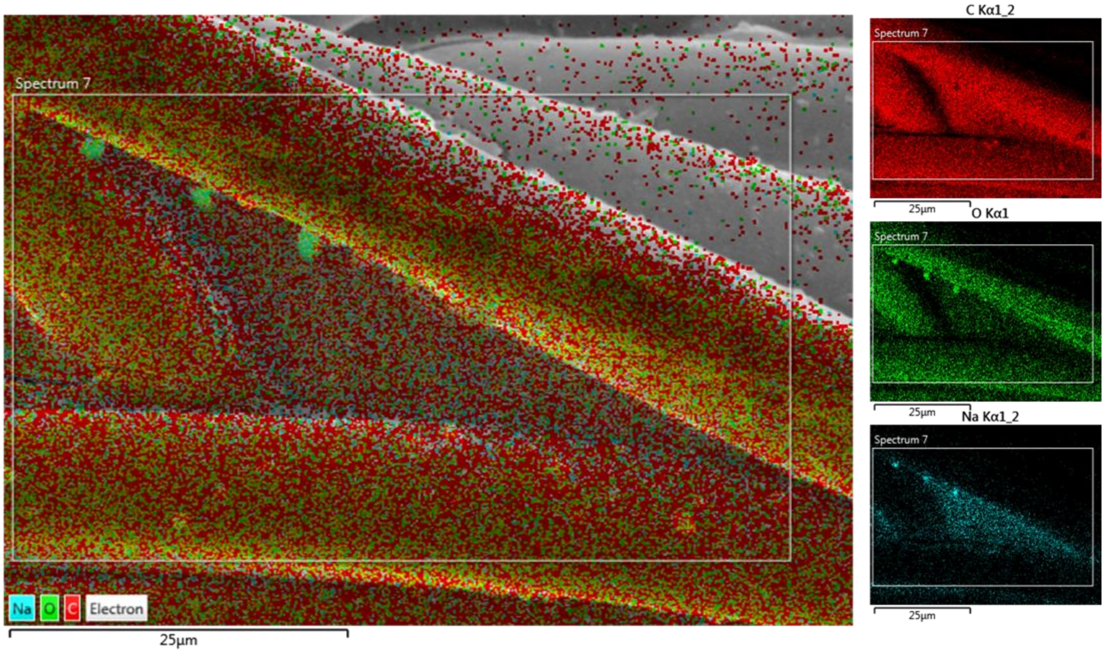

(c)

Figure S4: EDS is performed using Auriga 60 cross beam with exciting voltage of 20kV, (a) SEM micrograph of the carbon nanocomposite coated fabric representing the area for EDS analysis, (b) elemental composition primarily containing carbon and oxygen, 2.7% of sodium and small amounts (<1%) of calcium and aluminum and, (c) distribution of elements showing higher density of sodium is in nanocomposite coating rich area, likely due to the presence of sodium-based surfactants used in the dispersion.

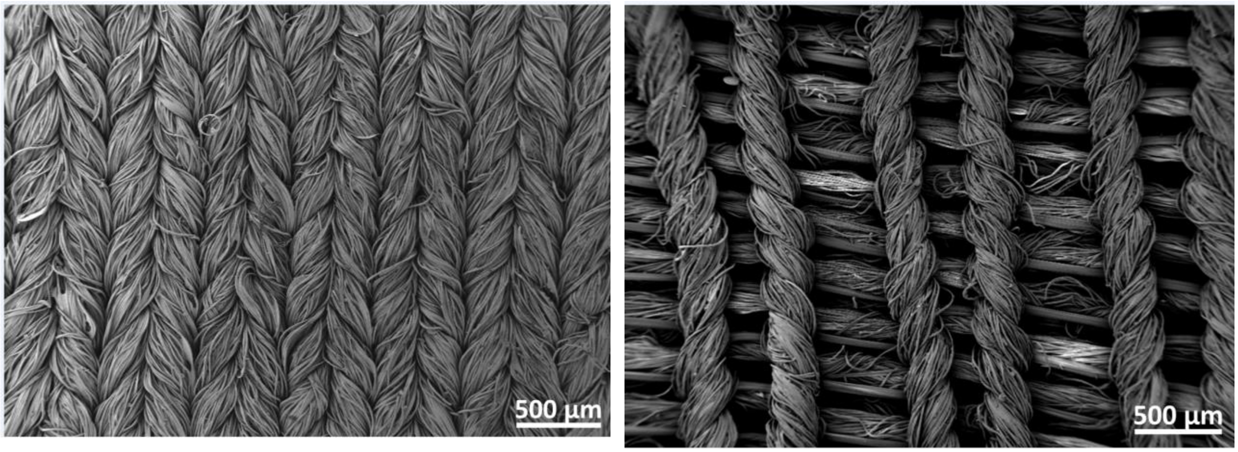

(a) (b)

Figure S5: SEM micrograph of carbon nanocomposite coated knit-fabric (a) unstretched and (b) bi-directional stretched

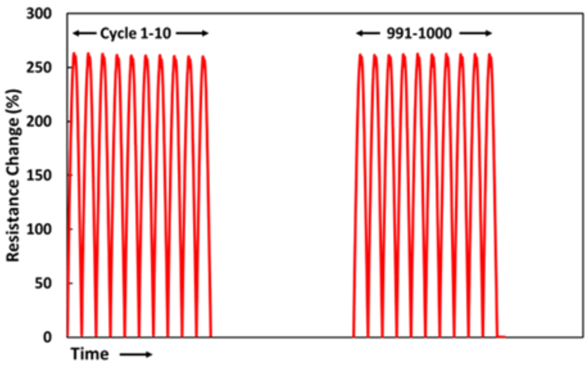

(a)

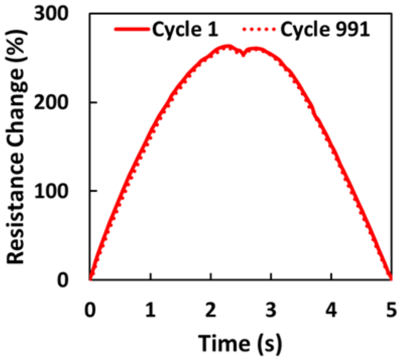

(b)

Figure S6: (a) Resistance change (%) in each cycle, in the first ten and last ten cycles in a 1000-cycle uniaxial tension test, showing no change in the overall sensor response, and (b) resistance change (%) with time for the first cycle and 991st cycle overlaid on the same time scale, showing no change in the individual cyclic sensor response.

Table S1: Resistance change during variable displacement straight line motion task, while the user is holding the Kinarm end point robot handle and motion is programmed

**Change in Angle (°)**	**Resistance Change (%)**					**Average Resistance Change (%)**	**Standard Deviation (%)**	**Coefficient of Variation**
	**Cycle 1**	**Cycle 2**	**Cycle 3**	**Cycle 4**	**Cycle 5**			
56	201.26	197.06	195.36	194.48	193.82	196.40	2.66	0.01
48	174.02	167.47	164.25	162.85	161.82	166.08	4.40	0.03
42	143.28	142.98	142.77	142.46	145.44	143.39	1.06	0.01
33	113.41	114.67	116.92	114.77	110.59	114.07	2.07	0.02
27	88.56	81.51	79.65	82.96	85.30	83.60	3.09	0.04
15	49.45	48.41	50.77	50.40	47.01	49.21	1.37	0.03

Table S2: Change in angle extrapolated from the resistance value measured during two-dimensional movement in diamond and circular paths for the sections 1-2, 2-3, 3-4 and 4-1

**Path**	**Section**	**Manual angle measured (°)**	**Change in Angle Extrapolated from Calibration Curve based on resistance value (°)**				**Average Change in Angle (°)**	**Standard Deviation (°)**	**Coefficient of Variation**
			**Cycle 1**	**Cycle 2**	**Cycle 3**	**Cycle 4**			
**Circular**	1-2	37	38.5	39	36	37	37.63	1.19	0.03
	2-3	20	20.5	18.5	19	17	18.75	1.25	0.07
	3-4	36	36	37	35	34	35.50	1.12	0.03
	4-1	19	23	22	20	20	21.25	1.30	0.06
**Diamond**	1-2	34	31	31	31	33	31.50	0.87	0.03
	2-3	22	23	25	23	24	23.75	0.83	0.03
	3-4	35	34	35	34	36	34.75	0.83	0.02
	4-1	20	20	21	20	21	20.50	0.50	0.02
